# Violet Light Transmission is Related to Myopia Progression in Adult High Myopia

**DOI:** 10.1038/s41598-017-09388-7

**Published:** 2017-11-06

**Authors:** Hidemasa Torii, Kazuhiko Ohnuma, Toshihide Kurihara, Kazuo Tsubota, Kazuno Negishi

**Affiliations:** 1 Department of Ophthalmology, Keio University School of Medicine, 35 Shinanomachi, Shinjuku-ku, Tokyo, 160-8582 Japan; 2 Laboratory of Photobiology, Keio University School of Medicine, 35 Shinanomachi, Shinjuku-ku, Tokyo, 160-8582 Japan; 3 Center for Frontier Medical Engineering, Chiba University, 1-33 Yayoi-cho, Inage-ku, Chiba, 263-8522 Japan

## Abstract

Myopia is increasing worldwide. Although the exact etiology of myopia is unknown, outdoor activity is one of the most important environmental factors for myopia control. We previously reported that violet light (VL, 360–400 nm wavelength), which is abundant in the outdoor environment, suppressed myopia progression for individuals under 20 years of age. However, whether VL is also effective for adult high myopia, which can be sight-threatening, has remained unknown. To investigate the influence of VL for adult myopia, we retrospectively compared the myopic progression and the axial length elongation over five years in adult high myopic patients over 25 years of age after two types (non-VL transmitting and VL transmitting) of phakic intraocular lens (pIOL) implantation. We found that high myopic patients with the non-VL transmitting pIOLs implanted are almost two times more myopic in the change of refraction and four times longer in the change of axial length, compared to those implanted with the VL transmitting pIOLs. This result indicated that the VL transmitting pIOL suppressed myopia progression and axial length elongation compared with the non-VL transmitting one. In conclusion, our study showed the VL possibly has an anti-myopia effect for human adults with high myopia.

## Introduction

The cause of the onset and progression of myopia (short-sightedness) is unknown, and the prevalence of myopia is increasing worldwide. If it continues to grow at its current rate, it is forecast that the world’s myopic population will be about 5 billion in 2050^1^. Myopia has been increasing worldwide, especially over the past 50 years^2^, and it seems that environmental changes are bigger factors than genetic changes.

Some epidemiological studies have suggested that increased near vision tasks such as reading, using computers and smartphones are possible risk factors^3^. Recently, the time spent outdoors was proposed as a protective factor^3–11^, and the beneficial effect of high ambient light for the protection of myopia has been confirmed in chicks, mice, and monkeys^12–16^. Additionally, some clinical trials on students have indicated that increased outdoor activity had an anti-myopia effect^11,17,18^. Furthermore, we recently discovered that violet light (VL, 360–400 nm wavelength), which hardly exists indoors and can only be found in outdoor environments, suppressed myopia progression^19^. According to the international lighting vocabulary of the Commission Internationale de l’Eclairage (CIE)^20^, the lower limits of visible light’s wavelengths are defined to be between 360 and 400 nm, which overlaps with the upper end of the Ultraviolet A (UVA) spectrum^21^. This range, in fact, is visible as VL, but it is recognized as UV as well. We compared the degree of myopia progression in two groups of students aged 13 to 18 years with different transmittance of VL in contact lenses used for refractive correction. We found that the axial length elongation in the group wearing VL transmitting contact lenses was smaller than those in the group wearing partially VL-blocking contact lenses, finding that axial length elongation was significantly less in the group wearing VL transmitting contact lenses^19^. However, these samples were younger students under 20 years old and not adults, and we did not know whether VL is also effective for adult high myopia, which can be sight-threatening in the future.

McBrien and Adams^22^ reported that the incidence of adult myopia development for two years in an occupational group aged from 21 to 63 years was 45%, and they confirmed the elongation of the vitreous chamber depth. Furthermore, Saka *et al*.^23^ reported that the axial length elongation of adult high myopic patients (myopia ≤ −6 diopters (D) or axial length ≥ 26.5 mm) whether there was a staphyloma or not, was confirmed in adults aged from 22 to 84 years.

Phakic intraocular lens (pIOL) implantation has been accepted as a refractive surgery for correcting high myopia^24,25^. Several designs have been developed, including angle-supported anterior chamber pIOLs, posterior chamber pIOLs, and iris-fixated pIOLs. Among them, the ARTISAN^®^ (Ophtec BV, Groningen, The Netherlands) and the ARTIFLEX^®^ (Ophtec BV) are iris-fixated pIOLs for which long-term results have been previously published^24,26–29^.

Here we show the difference in transmittance of VL between the ARTISAN^®^ and ARTIFLEX^®^ pIOL and compare the change of the refraction and axial length elongation over a long-term period between the two lenses. Then, we evaluate whether VL had the anti-myopia effect even for human adults with high myopia.

## Results

### Comparing the myopic progression and axial length elongation over five-year period retrospectively between the patients implanted with ARTISAN^®^ or ARTIFLEX^®^ pIOL against adult high myopia

We compared the change of refraction and axial length elongation for five years in high myopic adult patients (average over −10.0 D) after refractive surgery, implanted with pIOLs to correct high myopia.

Optical coherence tomography (OCT) confirmed that there were no cases of posterior staphyloma in both groups 5 years postoperatively.

**Figure 1:**
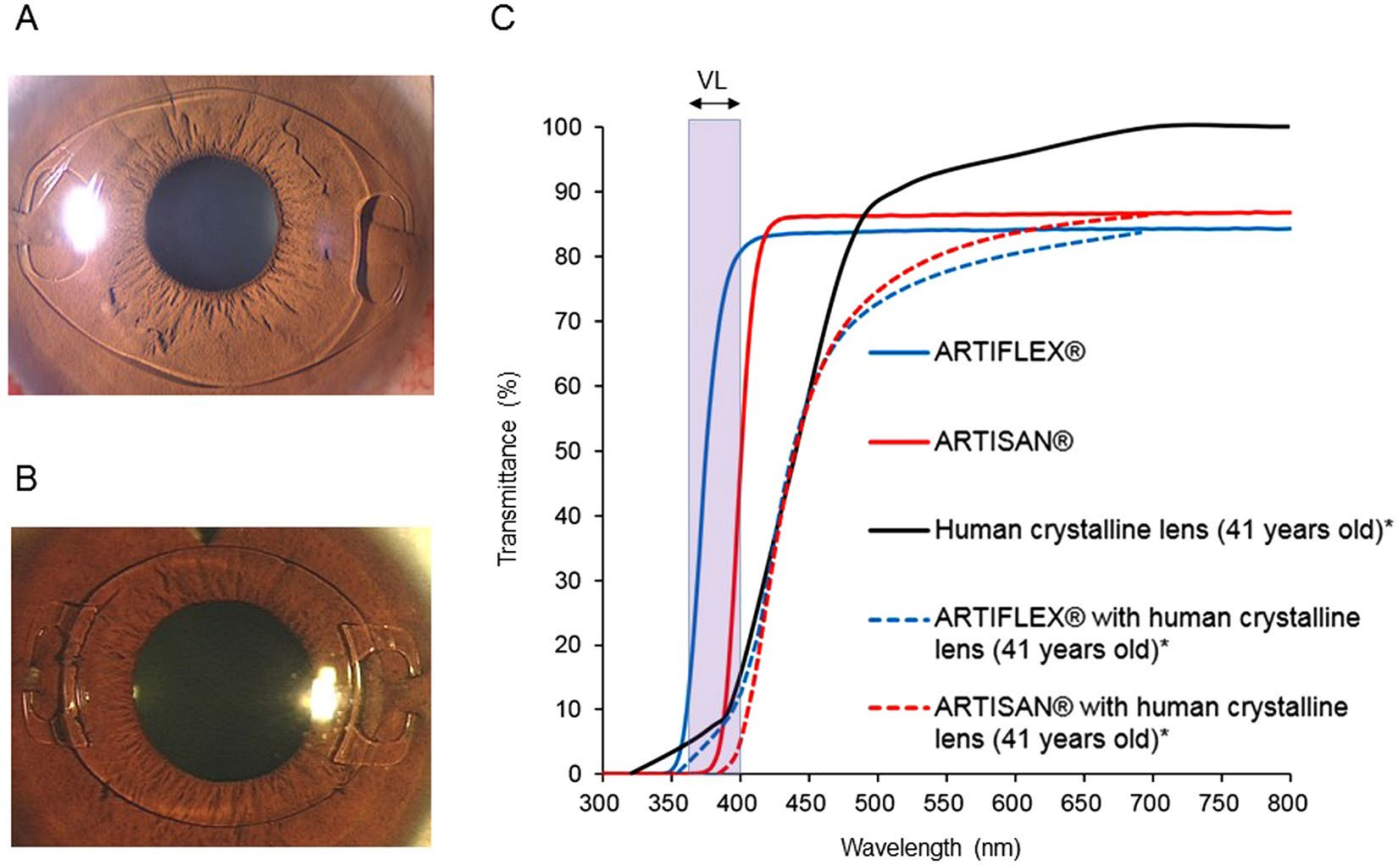
The two types of iris-fixated phakic intraocular lens (pIOL). (**A**) ARTISAN^®^ (Ophtec BV, Groningen, Netherlands) pIOL. (**B**) ARTIFLEX^®^ (Ophtec BV) pIOL. (**C**) Spectral transmission of ARTISAN^®^ and ARTIFLEX^®^ pIOLs. ARTISAN^®^ pIOL transmits minimal violet light (VL). ARTIFLEX^®^ is a VL transmitting pIOL. The calculated transmittance of ARTIFLEX^®^ or ARTISAN^®^ combined with human crystalline lens (41 years old)* was also shown. *Data from the spectral transmission of a 41-year-old human crystalline lens was taken from the figure of a published paper^31^, and the Association for Research in Vision and Ophthalmology is the copyright holder of this paper^31^.

**Figure 2:**
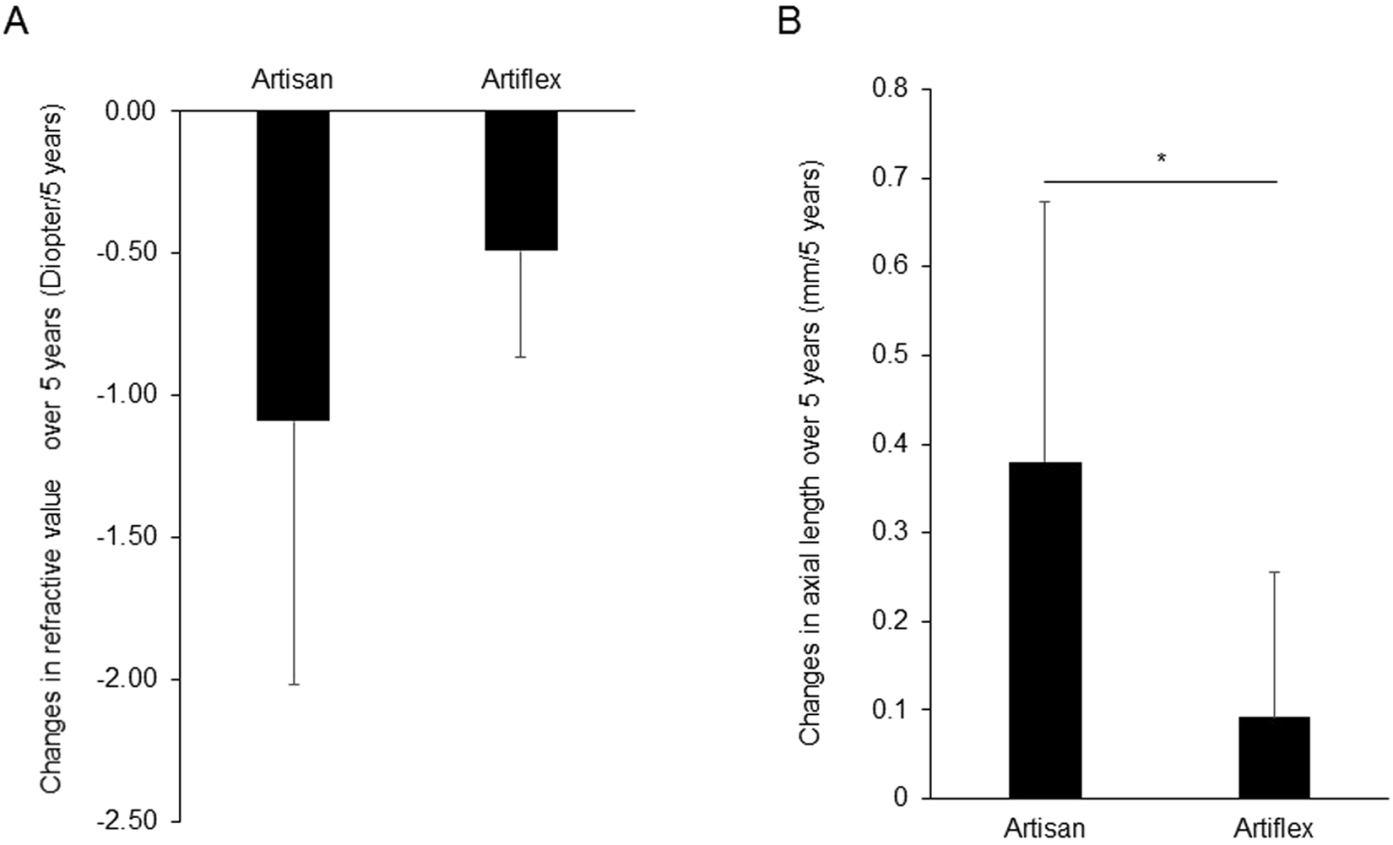
Violet light (VL) through eye suppressed myopia progression and axial length elongation in adult humans who were implanted with non-VL transmitting phakic intraocular lens (pIOL) and VL transmitting pIOL. (**A**) Changes in the refraction for about 5 years (5 years postoperatively minus 3 months postoperatively). The refractive values are more myopic in the Artisan group (11 cases of 11 eyes) than the Artiflex group (15 cases of 15 eyes). (**B**) Changes in axial length over 5 years (5 years postoperatively minus preoperative data). Changes in axial length in the Artiflex group (13 cases of 13 eyes) was significantly lower than the Artisan group (10 cases of 10 eyes). *P < 0.05, Mann-Whitney *U* Test. Data are shown as mean ± standard deviation (SD).

The mean change in the refraction after non-VL transmitting pIOL (11 cases of 11 eyes) (ARTISAN^®^, Fig. 1A,C) implantation was −1.09 D/5 years, whereas the change in the refraction after VL transmitting pIOL (15 cases of 15 eyes) (ARTIFLEX^®^, Fig. 1B,C) implantation was −0.49 D/5 years (P = 0.121) (Fig. 2A).

The mean axial length elongation after non-VL transmitting pIOL (10 cases of 10 eyes) (ARTISAN^®^) implantation was 0.38 mm/5 years, whereas the axial length elongation after VL transmitting pIOL (13 cases of 13 eyes) (ARTIFLEX^®^) implantation was only 0.09 mm/5 years (P = 0.030) (Fig. 2B).

There were no significant differences in preoperative age, cycloplegic refractive value, axial length, uncorrected visual acuity, or best corrected visual acuity between the two groups (Table 1). Table 2 shows the difference in some parameters including spherical aberration^30^ and the transmission of VL, 360–400 nm wavelength between the two lenses. These findings indicated that VL transmittance through pIOLs was possibly related to myopic progression in adult high myopia after pIOL implantation. Table 3 shows the results of all the individual subjects.

**Table 1:** Violet light (VL) exposure to the eye suppressed axial length elongation in adult humans: Comparing patients who were implanted with non-VL transmitting phakic intraocular lens (pIOL) or VL transmitting pIOL.

Parameter	VL (−) pIOLs	VL (+) pIOLs	*P* value
	Artisan group mean ± SD (range)	Artiflex group mean ± SD (range)	
Number	11 cases 11 eyes	15 cases 15 eyes	−
Race	All Japanese		
Age (years)	39.9 ± 8.9 (28 ~ 58)	36.3 ± 7.2 (26 ~ 53)	0.259
Cycloplegic refractive value (diopter) (Spherical equivalent)	−12.96 ± 4.19 (−18.13 ~ −6.88)	−11.14 ± 1.65 (−13.88 ~ −7.63)	0.318
Axial length (mm)	28.54 ± 1.85 (26.22~32.37)	28.13 ± 1.41 (25.30~30.53)	0.799
UCVA (logMAR)	1.48 ± 0.26 (0.90 ~ 1.90)	1.49 ± 0.14 (1.22 ~ 1.80)	0.878
BCVA (logMAR)	−0.11 ± 0.16 (−0.30 ~ 0.30)	−0.15 ± 0.12 (−0.30 ~ 0.15)	0.721

Patient preoperative data: Artisan group (n = 11), the phakic intraocular lens (pIOL) group who were implanted with ARTISAN^®^ pIOLs [VL (−) pIOLs]; and the Artiflex group (n = 15), the pIOL group who were implanted with ARTIFLEX^®^ pIOLs [VL (+) pIOLs]. There were no significant differences in preoperative age, cycloplegic refractive value, axial length, uncorrected visual acuity (UCVA), and best corrected visual acuity (BCVA) between the two groups. SD, standard deviation. Data were analyzed using the Mann-Whitney *U* Test.

**Table 2:** Difference in parameters between ARTISAN^®^ and ARTIFLEX^®^ pIOLs: The most notable point is the difference of transmission of VL, 360–400 nm wavelength between the two lenses.

	ARTISAN^®^	ARTIFLEX^®^
Spectral transmission	VL blocked	Not VL blocked
Incision size during operation (mm)	6.5	3.2
Refractive index	1.49	1.43
Lens material of optical zone	Polymethyl methacrylate (PMMA)	Silicone
Spherical aberration (Z_4_^0^)	—	Lower than ARTISAN^®^*

*Spherical aberration (Z_4_^0^) is not measured by the manufacturer, but based on the ref.^30^. pIOL, phakic intraocular lens. VL, violet light.

**Table 3:** All the individual subject’s results.

Group	Age	Changes in the refraction for approximately 5 years (5 years postoperatively minus 3 months postoperative data) (Diopter)	Changes in axial length over 5 years (5 years postoperatively minus preoperative data) (mm)
Artisan group	58	−1.38	NA
	46	0.25	0.33
	34	0.00	0.08
	28	−1.50	0.61
	38	−1.50	0.65
	44	−2.88	0.69
	31	−1.38	0.67
	32	−0.13	0.00
	46	−1.50	0.03
	46	−0.38	0.14
	36	−1.63	0.60
Average of Artisan group	39.9	−1.09	0.38
Artiflex group	31	−0.88	0.31
	37	−1.00	NA
	42	−0.26	NA
	41	0.25	0.25
	36	−0.63	−0.04
	33	−1.00	0.13
	42	−0.50	−0.26
	36	−0.38	0.01
	44	−0.75	−0.04
	26	−0.75	0.18
	28	−0.25	0.08
	34	−0.63	0.10
	53	−0.25	0.15
	33	−0.50	−0.02
	28	0.13	0.34
Average of Artiflex group	36.3	−0.49	0.09

NA = Not Applicable.

### Higher order aberrations (HOAs) and residual astigmatism

There were no significant differences in HOAs and residual astigmatism 5 years postoperatively between the two groups (Table 4).

**Table 4:** Comparisons of residual astigmatism, and higher order aberrations between the Artisan and Artiflex groups 5 years postoperatively.

	Artisan Group (mean ± SD)	Artiflex Group (mean ± SD)	*P* Value
Number	11 cases 11 eyes	15 cases 15 eyes	−
Residual astigmatism (diopter)	−0.68 ± 0.53	−0.55 ± 0.40	0.474
J0 component of residual astigmatism (diopter)	0.10 ± 0.28	0.01 ± 0.27	0.198
J45 component of residual astigmatism (diopter)	0.11 ± 0.30	0.02 ± 0.22	0.357
Cornea THOA (µm)*	0.05 ± 0.02	0.05 ± 0.03	0.643
Internal THOA (µm)*	0.07 ± 0.03	0.06 ± 0.02	0.238
Ocular THOA (µm)*	0.08 ± 0.04	0.06 ± 0.02	0.216

SD = standard deviation; THOA = total higher-order aberrations, root mean square (RMS) of the third- to sixth-order Zernike coefficients. *Pupillary diameter = 4 mm (Artisan group [10 cases 10 eyes], Artiflex group [15 cases 15 eyes]). Data were analyzed using the Mann-Whitney *U* Test.

### Simulation of the peripheral refraction after pIOL implantation

**Figure 3:**
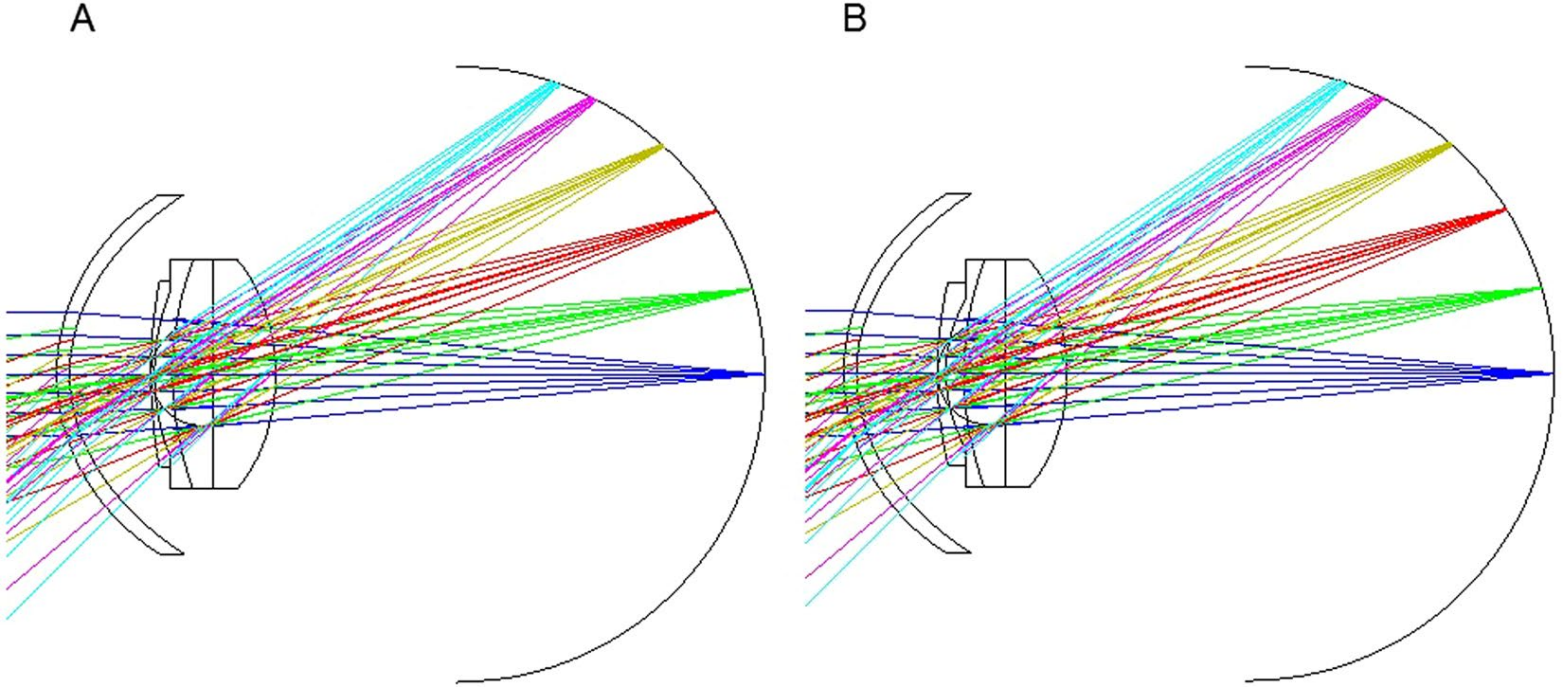
Simulation results of peripheral refraction after ARTISAN^®^ and ARTIFLEX^®^ phakic intraocular lens (pIOL) implantation. (**A**) Simulation result of peripheral refraction after ARTISAN^®^ pIOL implantation. (**B**) Simulation result of peripheral refraction after ARTIFLEX^®^ pIOL implantation. There is no difference in the amount of peripheral hyperopic defocus between ARTISAN^®^ and ARTIFLEX^®^ in the eye model simulation. This indicates that the differences in myopic progression and axial length elongation between the two pIOLs did not seem to depend on the difference of peripheral hyperopic defocus.

The focusing images were nearly identical. Even if the wavelength is around 400 nm, the focusing images showed the same results as Fig. 3A,B. The defocus amounts were the same and +1.18 D at the incident angle of 20 degrees for the two pIOLs. They were +2.93 D for ARTISAN^®^ pIOL, and +3.04 D for ARTIFLEX^®^ pIOL, at the incident angle of 30 degrees.

## Discussion

First, we measured the transmittance of the ARTISAN^®^ and ARTIFLEX^®^ pIOLs, and we confirmed that ARTISAN^®^ was a non-VL transmitting pIOL, ARTIFLEX^®^ was a VL transmitting pIOL (Fig. 1C). Then, we retrospectively studied human clinical data and found that VL also had the suppressive effect against adult (>25 years old) high myopia as well as school-aged (<20 years old) myopia^19^. It is notable that high myopic patients who were implanted with the non-VL transmitting pIOLs were almost twice as myopic in the change of refraction and four times longer in the change of axial length and more likely to suffer from myopia progression, compared to those who were implanted with the VL transmitting pIOLs. We demonstrated that the VL transmitting pIOL suppressed myopia progression and axial length elongation compared with the non-VL transmitting one.

The changes of the axial length in adult high myopia without staphyloma (the average age was 46.5 years of age and age range was from 22 to 76 years of age, axial length ranged from 26.58 to 33.17 mm, which is almost the same as our current study) was 0.06 mm/year^23^ and the changes of the axial length in an adult high myopia with staphyloma (the average age was 52.4 years and age range was from 29 to 84 years old, axial length ranged from 26.45 to 34.43 mm) was also 0.06 mm/year^23^. The history of refractive surgery was one of the exclusion criteria in that study. Therefore, the patients were assumed to have used contact lenses or eyeglasses for the refractive correction, although the details of myopic correction method were not described in the paper. According to our study^19^ on the spectral transmission of the most commonly used eyeglasses and contact lenses in Japan, almost all eyeglasses blocked VL, and some contact lenses blocked VL partially, although the other contact lenses were transmitting VL. In the current study, the group of non-VL transmitting pIOLs (ARTISAN^®^; Artisan group) showed the greater axial length elongation (0.38 mm/5 years = 0.076 mm/year) than the group of VL transmitting pIOLs (ARTIFLEX^®^; Artiflex group) (0.09 mm/5 years = 0.018 mm/year). Comparing this data with the previously reported ones^23^ for the patients with the almost the same background, the group of contact lenses or eyeglasses showed the axial length elongation (0.06 mm/year) in between the non-VL transmitting and the VL transmitting pIOLs. These results are understandable for the transmittance of VL, though this comparison was examined among different materials as well as different spectral transmissions.

In the next step, we considered whether VL passes through the crystalline lens to the retina in order to induce the suppressive effect against myopia progression. We calculated the VL transmittance of a 41-year-old human crystalline lens from a previous study^31^, which is similar to the age bracket of the subjects in the current study, resulting in approximately 10% of VL being transmitted through the human crystalline lens. This data suggested that the VL spectral transmittance reaching the retina would be different between the Artisan and Artiflex groups even though VL transmission is reduced by the crystalline lens. Although we speculated that chorioretinal tissue could be one of the targets of VL exposure^19^, other tissues, including the crystalline lens, should also be considered. Further investigation on the target tissues of VL in addition to the choroid and retina should be conducted in the future.

Refractions in the peripheral retinal area have been considered a controversial issue for myopia progression^32–34^ and shapes of the eye differ by individual. We simulated the refractions in the peripheral retinal area in the same eye model (Fig. 3A,B). However, we found no differences in peripheral hyperopic defocus between the two pIOLs. Though we did not measure the real peripheral hyperopic defocus of these patients, these simulation results implicated that the differences in myopic progression and axial length elongation between the two pIOLs did not depend on the difference of the peripheral hyperopic defocus.

There are some limitations in the current study. First, the sample size is small though it is estimated sufficient according to the statistical assumption. Second, the differences in factors other than the spectral transmittance, such as postoperative pupil diameter, refractive index, lens material, and spherical aberration^30^ between the two pIOLs (Table 2) were not evaluated. These parameters should be investigated in the future. Third, other confounding factors in myopia such as outdoor time^7,8,17^, time spent in near work^35^, and parental myopia^36^ were not included in the current study. Further investigation is being considered on those confounding factors in our future studies. Fourth, the ARTISAN^®^ pIOL was indicated when the anterior chamber depth was from 2.8 to 3.2 mm (7 out of 11 cases), which is one of the selection biases in the current study.

In summary, though there are no effective methods to retard adult high myopia and axial length elongation, the introduction of VL exposure may contribute to the reduction of myopic progression and axial length elongation even for adult humans with high myopia. Violet light may be one of the potent candidates for prevention of myopia, especially for sight-threatening high myopia.

## Methods

### Comparison in the myopic progression and axial length elongation of adult human between the non-violet-light transmitting pIOL (ARTISAN^®^) group and the violet light transmitting pIOL (ARTIFLEX^®^) group

We retrospectively evaluated the change of refraction and the axial length elongation after iris-fixated pIOL implantation and compared them between two pIOLs, ARTISAN^®^ (Ophtec BV) and ARTIFLEX^®^ (Ophtec BV) (Fig. 1A,B). The Keio University School of Medicine Ethics Committee approved the clinical study. All procedures involving human subjects were performed in accordance with the tenets of the Declaration of Helsinki. Written informed consents were obtained from all patients after they received the explanation. The ARTISAN^®^ pIOL has a convex-concave polymethyl methacrylate (PMMA) optic with a 6.0-mm optical zone and PMMA haptics. The ARTIFLEX^®^ pIOL has a convex-concave silicone optic with a 6.0-mm optical zone and PMMA haptics. The refractive indices were 1.49 for the ARTISAN^®^ pIOL and 1.43 for the ARTIFLEX^®^ pIOL. The ARTISAN^®^ pIOL transmits minimal VL whereas the ARTIFLEX^®^ pIOL transmits more VL (Fig. 1C). Twenty-six eyes of 26 Japanese patients with myopia exceeding −6.00 D were followed for over a five-year period after pIOL implantation at Keio University Hospital. One experienced surgeon (K.N.) performed all pIOL implantations. They were divided into two groups: the Artisan group, the pIOL group with 11 eyes of 11 patients (mean age, 39.9 ± 8.9 years) implanted with ARTISAN^®^ pIOLs; and the Artiflex group, the pIOL group with 15 eyes of 15 patients (mean age, 36.3 ± 7.2 years) implanted with ARTIFLEX^®^ pIOLs. The primary outcome measures were the differences in the refraction and axial length elongation over a five-year period after implantation of the pIOLs between the two groups. The patient preoperative data is shown in Table 1. There were no significant differences in preoperative age, cycloplegic refractive value, axial length, uncorrected visual acuity and best-corrected visual acuity between the two groups. The inclusion criteria for both groups were a minimum age of 25 years with myopia exceeding −6.00 D; no previous ocular surgery, uveitis, cataracts, diabetic retinopathy, corneal diseases, glaucoma, or a history of ocular trauma. The inclusion criteria for pIOL implantation was a sufficiently deep anterior chamber (2.8 mm or more for the ARTISAN^®^ pIOL and 3.2 mm or more for the ARTIFLEX^®^ pIOL), and an adequate endothelial cell count (2,000 cells/mm^2^ or higher). Regarding the selection of a pIOL, the ARTISAN^®^ pIOL was indicated when the anterior chamber depth was from 2.8 to 3.2 mm (7 out of 11 cases). When both pIOLs were indicated, the patients selected the pIOL after they received an explanation of the differences in the incision size, lens materials, long-term results, and complications between the ARTISAN^®^ and ARTIFLEX^®^ pIOLs. The exclusion criteria for both groups were active ocular or systemic disease likely to affect wound healing, pregnancy, and nursing mothers. No intraoperative or postoperative complications developed and no enhancements were performed. Objective refraction was measured by autorefractor (ARK-700A, NIDEK) five times, and we used the mean data for analysis. The axial length was measured five times preoperatively and postoperatively using the phakic mode of the IOLMaster (Carl Zeiss Meditec), and we used the mean data for analysis. Surgical technique and preoperative and postoperative treatment were the same as previously described^37^.

### Spectral transmission of pIOLs

The UV-visible absorption spectra of these samples were recorded with a UV-2600 spectrophotometer (Shimadzu Corporation, Kyoto, Japan) (Fig. 1C).

### HOAs and residual astigmatism

Each measurement of the corneal, internal, and ocular HOAs were recorded 5 years post-operatively using the OPD-Scan (Nidek Co., Aichi, Japan). The most reliable data (i.e., the well-delineated line of the pupillary edge, clear Placido rings, and the absence of the eyelid edge on the cornea in the map of the eye image) were adopted as the final results based on the image quality. Corneal HOAs originate from the anterior cornea; internal HOAs originate from the lens, the pIOL, and the posterior corneal surface. All wavefront aberrations were calculated and plotted with respect to the corneal vertex. The corneal topography was measured using Placido disk technology, and the ocular wavefront was measured using the principle of skiascopic phase difference.

The coefficients of Zernike polynomials were determined up to the sixth order for 4-mm optical zones from the wavefront data. All measurements were performed under maximal mydriasis induced by eye drops containing 0.5% tropicamide and 0.5% phenylephrine hydrochloride (Mydrin-P; Santen Pharmaceutical Co., Osaka, Japan); the pupillary diameters during measurements exceeded 4 mm in all patients. The root mean squares (RMSs) of the total HOAs from the third- to sixth-order Zernike coefficients were calculated.

Residual astigmatism was the subjective astigmatism 5 years postoperatively. The astigmatism vector was decomposed into vertical/horizontal (J0) and oblique (J45) components using the power vector analysis described by Thibos *et al*.^38^. The conversion to power vector notation was performed using the following equations^38^, where C is the negative cylindrical power and α is the cylinder axis: J0 = (−C/2) cos (2α), J45 = (−C/2) sin (2α).

### Simulation of peripheral refraction after pIOL implantation

The focusing images of the pIOLs on the peripheral parts of the retina were calculated using Liou-Brennan eye model^39^ and ZEMAX optics design software (Radiant Zemax, Washington, USA). The paraxial focus position of the pIOLs with −10 D power was obtained and was 23.05 mm from the back surface of the crystalline lens at the wavelength of 546.1 nm. The retinal spherical surface with the radius of −12.0 mm was set and the defocus amounts for the incident angles of 0, 10, 20, 30, 40, 45 degrees were calculated at the pupil of 4 mm diameter. Each focus is presented by the seven light rays as shown in Fig. 3A,B.

### Statistical analysis

A Mann-Whitney *U* Test was used to compare data between the two groups for the clinical study. A *P* value of less than 0.05 was considered significant. All statistical analyses were performed using IBM SPSS Statistics version 21.0 (IBM Corp, New York, USA).

The sample size calculation indicated that a sample size of at least 8 cases would be required (a minimum acceptable reduction of a difference of 0.25 mm over the five-year period, so 8 cases in each group were needed to have a 5% alpha level and 95% power).
